# “The whole sky has broken down on me. I might die alone”: A qualitative study on the lived experiences of COVID-19 positive frontline workers in Bangladesh

**DOI:** 10.3389/fsoc.2022.1054921

**Published:** 2022-11-16

**Authors:** Shamsul Arefin, Tamanna Rashid, Mowsume Bhattacharjee, Md. Didarul Habib, Md. Ashraful Islam, Mohammad Anisur Rahaman

**Affiliations:** ^1^Department of Sociology, Bangabandhu Sheikh Mujibur Rahman Science and Technology University, Dhaka, Bangladesh; ^2^Department of Sociology, Jagannath University, Dhaka, Bangladesh; ^3^School of Sociology, Central China Normal University, Wuhan, China; ^4^Department of Sociology, University of Dhaka, Dhaka, Bangladesh; ^5^A2i-Aspire to Innovate, A Bangladesh Government and UNDP Supported Programme, Dhaka, Bangladesh; ^6^School of Public Affairs, Zhejiang University, Hangzhou, Zhejiang, China

**Keywords:** COVID-19, frontline Workers, phenomenology, Colaizzi method, lived experiences, Bangladesh

## Abstract

Many countries, including Bangladesh, have conducted research on the mental health of frontline workers and their challenges in adjusting to their new workplaces. However, the authors are unaware of any studies on their real-life experiences as COVID-19-positive patients in Bangladesh. This study intends to investigate the lived experiences of Bangladeshi frontline workers who were isolated as a result of the COVID-19 infection and tested positive for the virus. We used a qualitative methodology and a semi-structured interview guide to conduct ten interviews between July 26 and August 12, 2020. The participants were recruited *via* a social media campaign and purposive sampling. All interviews were conducted *via* telephone and online and were transcribed and analyzed using Colaizzi's phenomenological method. The study does, however, identify four primary themes and 13 supporting themes, including (1) experience in a new working environment (subthemes: workload and adaptation, maintaining health protocol and social distance, and the fear of infection), (2) diagnosis (subthemes: the origin of infection, physiological problems, experiences at the diagnosis center), (3) recovery days (subthemes: earlier reactions, experiences in isolation, coping mechanisms), and (4) post-COVID-19 (subthemes: excitement, fear, and confusion; physiological problems; increased religiosity; and changes in philosophy). This study is important for healthcare policymakers because it helps them design healthcare management systems that take Bangladeshi society's social context into account. This study also recommends that long-term behavioral change programs be implemented by national policymakers to lessen societal stigma. At the same time, it suggests that the government should help lessen the barriers to health care services that persons with lower socioeconomic status confront.

## Introduction

The SARS-CoV-2 (Severe Acute Respiratory Syndrome Coronavirus 2) virus, which causes COVID-19, is highly contagious and pathogenic. It has sparked a global pandemic that has resulted in a large number of deaths around the world. Although there is debate about the exact origin and source of the virus, Wuhan- an emerging economic and commercial hub in China initially saw an outbreak of a new coronavirus by the end of 2019 that killed over 1,800 people and infected over 70,000 individuals in the first 50 days of the COVID-19 epidemic. In 2003 a viral infection caused by the SARS coronavirus broke out in southern China's Guangdong province (Shereen et al., 2020), which created a pandemic that affected 26 countries worldwide and claimed more than 8,000 infections and 776 deaths (World Health Organizaition, 2003). However, SRAS-CoV (2003) had a fatality rate of 9 percent (Shereen et al., 2020), whereas SRAS-CoV-2 (2019) has infected 619,836,103 people in 228 countries and territories with 6,539,363 deaths (Worldometer, September 24, 2022), indicating that SARS-CoV-2 has an even greater transmission rate than SRAS-CoV.

Bangladesh officially declared its first identified COVID-19 case on March 8, 2020, and confirmed the first death on March 18, 2020, which puts us at 2,020,768 confirmed cases and 29,347 deaths until January 2022 (Worldometer, September 24, 2022). According to the Bangladesh Medical Association, about 8,890 frontline healthcare workers tested positive among the infected, and 87 died (Rosenvald, 2022). Besides the healthcare workers, frontline workers, including 833 journalists, 8,331 police officers, and 150 administrative workers, were identified as COVID-19 positive; around 60 died among them (New Age, June 19, 2020; Rosenvald, 2022). The countless frontline workers who continue to work despite the adversities and health hazards associated with the COVID-19 outbreak are the true heroes of our time. They work very hard amidst the severity of the pandemic to keep people safe and healthy. Global evidence also reports that the number of infections among frontline workers, especially healthcare providers, is higher than in general people (Liu Y., et al., 2020). In addition, due to the nature of work frontline workers worldwide suffer from various psychological and physiological problems (Lai et al., 2020; Shahbaz et al., 2021). Several studies have shown a high prevalence of mental health symptoms, such as anxiety, insomnia, distress, and psychological burden among frontline healthcare workers while treating patients with COVID-19 (Muller et al., 2020; Upadhyaya et al., 2020; Wang et al., 2020; Sun et al., 2021; Zhang et al., 2021). Studies also revealed that frontline workers faced various professional and psycho-social challenges during this pandemic that included a harsh working environment, fear of contaminating family members and others, adaptation to continuously changing guidelines, and a lack of PPE (Lai et al., 2020; Hossain M. A, et al., 2021; Khatun et al., 2021; Shahbaz et al., 2021). Moreover, COVID-19-positive frontline workers have been seen to hide their symptoms from their loved ones because of the fear of stigmatization (Logie and Turan, 2020; Kwaghe et al., 2021).

Numerous studies are being undertaken throughout the world to examine the psycho-social experiences of frontline workers caring for COVID-19 patients during this pandemic (Lai et al., 2020; Aughterson et al., 2021; Dagyaran et al., 2021; De Kock et al., 2021; Deng and Naslund, 2021; Magner et al., 2021; Nguyen et al., 2021; Sun et al., 2021; van der Goot et al., 2021; Wang et al., 2021, 2022; Yin et al., 2022). Nevertheless, a few global studies describe COVID-19-positive frontline workers' lived experiences (Fawaz and Samaha, 2020; Siagian and Rantung, 2022; Simeone et al., 2022). For example, Siagian and Rantung (2022) explored the lived experiences of seven Indonesian healthcare nurses who tested positive for COVID-19 in a descriptive phenomenological study. They recognized the pre-isolation, isolation, and post-isolation lived experiences of survivors in their study, which included, among other things, fear, reaction, feelings in the isolation room, and post-COVID-19 condition. Similar to this, De Simone et al. (2022) examined the lived experiences of Italian frontline nurses and doctors who were affected by COVID-19. They looked at the strong emotional impact of COVID-19 on nurses and doctors who contracted it while performing their duties, including feelings of fear and loneliness on the one hand and impotence and guilt for not being able to help on the other. However, prior to this study, there was a dearth of literature in Bangladesh about the lived experiences of front-line healthcare workers who tested positive for COVID-19.The majority of COVID-19 studies in Bangladesh have concentrated on the work experiences of frontline workers, their mental health problems, and associated stigma in healthcare settings and outside (Farhana, 2020; Akhter et al., 2021; Hossain M. A, et al., 2021; Hossain M. B, et al., 2021; Khan Rony et al., 2021; Khatun et al., 2021; Rahman et al., 2021; Rana and Islam, 2021; Razu et al., 2021; Sakib et al., 2021; Mehedi and Ismail Hossain, 2022; Miah et al., 2022; Pooja et al., 2022; Rahman, 2022; Tune et al., 2022; Uddin et al., 2022). In addition, a group of researchers in Bangladesh conducted a telephone-based survey of 322 healthcare professionals to examine their quality of life after being cured of coronavirus (Rashid et al., 2022).

Moreover, prior COVID-19 research did not examine the lived experiences of various infected frontline workers, including healthcare providers, law enforcement personnel, journalists, and bankers. The current study will address this knowledge gap by examining the experiences of Bangladeshi frontline workers who tested positive for COVID-19. Moreover, as individual viewpoints might vary depending on a person's culture and society, it is crucial to explain and comprehend frontline workers' experiences in a particular context. Additionally, their untold stories about their work, diagnosis, isolation, socio-psychological vulnerabilities, social support, and organizational support throughout and after their quarantine will help policymakers and stakeholders design efficient strategies to lessen their work-related burden and discrimination in Bangladesh.

## Materials and methods

### Research design

Using Colaizzi's phenomenological method we qualitatively analyzed the lived experiences of frontline workers in Bangladesh who were identified as COVID-19-positive patients. This approach seeks to comprehend people's daily experiences. It identifies common patterns of meaning rather than unique characteristics in the research subjects, ensuring the validity of the information gathered from the participants (Shosha, 1857; Sanders, 2003).

### Participants

From July 26, 2020, to August 12, 2020, we selected ten COVID-19 positive frontline workers (three medical frontline workers and seven non-medical frontline workers) from various sources, including a social media campaign and personal contact. We adopted the required number of participants by interviewing those who met the following inclusion criteria until the data was saturated and no new themes were generated.

The inclusion criteria included:

1. Of those who had contracted COVID-19 after a diagnosis,
2. Those who had undergone at least a 2-week isolation independently or in a hospital, and
3. Those who had formally agreed to participate in the study after being informed of its goals.

### Interview outline

We developed the interview outline after reviewing pertinent literature and getting feedback from group members at numerous meetings. The interview subjects were asked the following questions: (1) What was your earlier life before infection? (2) How did you come to know about your infection? (3) What were your initial responses after identifying yourself as a COVID-19 patient? (4) What were your experiences during the isolation days? (5) How did you cope with this situation? (6) What aspects of life changed after your recovery from COVID-19?

### Data collection

An email including a consent form and an interview guide was sent to participants who indicated an interest in participating in this study. After the participants gave their consent, an interview was scheduled and held whenever it suited them. The five research team members (SA, TR, MB, MH, and MI) conducted each interview. Due to the pandemic, the interviews were carried out over the telephone and on online platforms (zoom, google meet). Each interview lasted 35–60 min and was conducted in Bangla. With the participants' consent, all interviews were audio recorded.

### Data analysis

Within 24 h of each interview, the team translated the audio recording from Bangla to English. The first two authors (SA and TR) then read each transcript several times to identify significant statements. Next, they took the meaning units from these statements and provided codes. After creating a preliminary list of themes that emerged from the diversity of transcripts, the connections between themes (cluster meaning) were established. The research team then engaged in frequent online meetings to extrapolate themes and subthemes from the coding. Finally, divergent opinions on the content of the topics were addressed and resolved by a professor with experience in qualitative research. We also conducted another interview with the participants to share the study's findings for validation.

### Ethics

This study followed the ethical agreement of the Helsinki declaration. The study participants had the right to withdraw from the research at any time. In gathering the data we maintained objectivity and built strong bonds with the individuals. Each participant's name was changed to a pseudonym to protect their identity. We kept all the information in a password-protected Google Drive storage system that was only available to us.

## Results

### Characteristics of the participants

Our study comprised three medical frontline workers and seven non-medical frontline workers who had been diagnosed as COVID-19 patients and were between the ages of 28 and 45. Their average age was 34 years. Three of them (30%) provided healthcare services, three (30%) worked in law-enforcement agencies and bureaucracy, and the remaining four (40%) worked in the financial sector and print media industries. Moreover, 30% of respondents were single, whereas 70% were married. In addition, seven respondents received treatment at home, while three others were admitted to COVID-19-dedicated hospitals. The duration of treatment lasted between 14 and 32 days. The participant's characteristics are outlined in Table 1.

**Table 1:** Characteristics of participants.

**Code**	**Gender**	**Age**	**Occupation**	**Marital status**	**Isolation**
R1	M	28	Sub-Inspector	Single	22
R2	F	31	Magistrate	Single	14
R3	M	45	Banker	Married	18
R4	M	38	Doctor	Married	14
R5	F	35	Banker	Married	14
R6	M	32	Doctor	Married	18
R7	M	35	Journalist	Married	32
R8	M	30	Banker	Married	21
R9	M	37	Doctor	Married	14
R10	M	28	Sub-Inspector	Single	16

**Figure 1:**
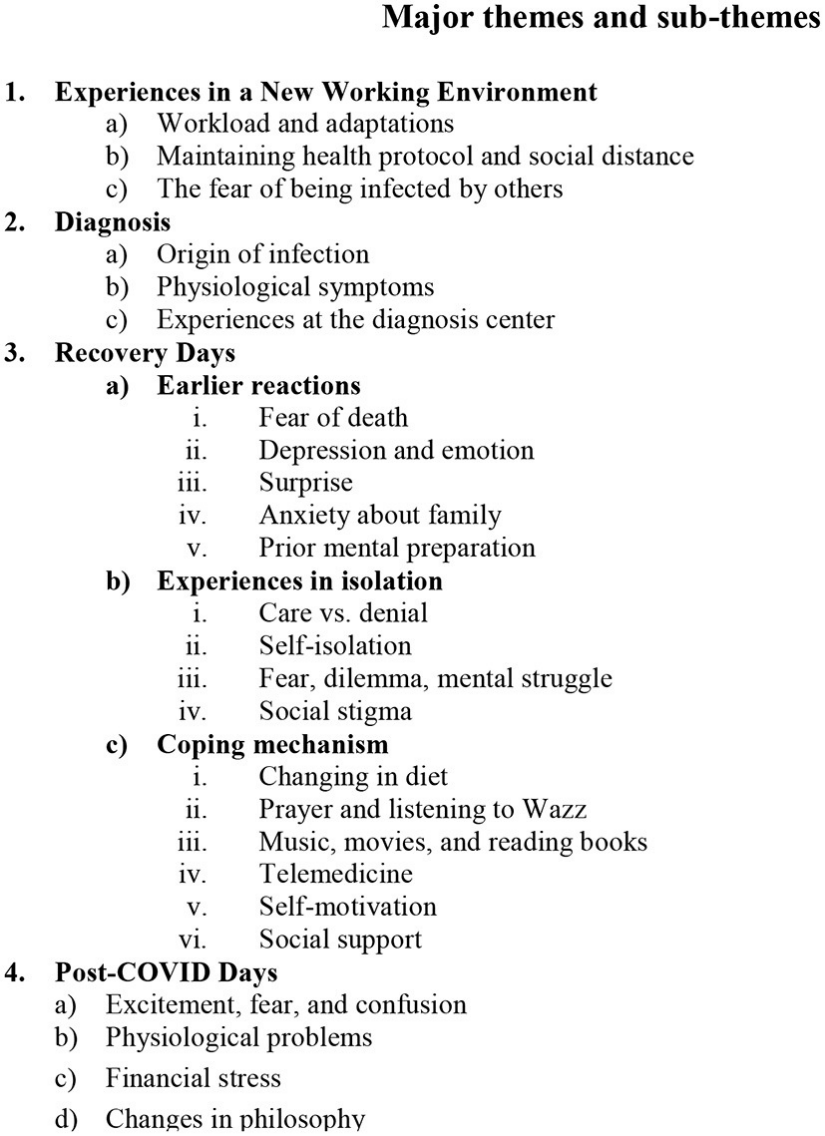
Major themes and sub-themes.

However, we analyzed the lived experiences of COVID-19-positive frontline workers in Bangladesh using phenomenological techniques. Four significant themes and 13 subthemes emerged from Figure 1. The analysis of the participant's responses is outlined below.

### Theme I: Experiences in a new working environment

Frontline workers in the COVID-19 situation encountered numerous challenges in a new work situation that impacted their physical, psychological, and social wellbeing. This theme consisted of three subthemes: workload and adaptations; maintaining health protocol and social distance, and the fear of being infected by others.

#### Workload and adaptations

None of the frontline workers in this study had worked in a pandemic environment like the current COVID-19 pandemic in Bangladesh. Therefore, they had to adapt to the new working situations and policies. Specially, medical frontline workers had to face dire conditions due to their direct involvement in treating COVID-19 patients in the corona unit. In addition, because of the increasing number of COVID-19 patients in Bangladesh, non-medical frontline workers were deployed and assigned to different places outside their routine work.

“*Since I am a government employee, I had to go to different places to perform official duties at the beginning of the corona infection in Bangladesh. I started working to ensure social distancing from the period when the government announced that no more than 10 or 15 people could gather in a place. Control of public gatherings at various institutions, wedding houses, religious and social functions, to ensure the home quarantine and isolation of expatriates and those who came from outside the district, etc., were my prime official duties during that time*” (R2).

“*I have been on the field since the beginning of Corona, highlighting the plight of patients in hospitals, including the mismanagement of sample collections, the neglect of unsuspected people, and the corporate propaganda called ‘corona drug.' While other stations shut down their crime investigation programs, Searchlight did not shut down for a single episode*” (R7).

#### Maintaining health protocols and social distance

Most of the frontline workers in this study expressed that they always tried to follow the health rules of the Directorate General of Health Services (DGHS) and World Health Organization (WHO) properly. Initially, medical frontline healthcare workers in Bangladesh had shortages of personal protective equipments and they had to work long hours in COVID-19 dedicated isolation units with corona patients. So maintaining health protocols were challenging at the beginning. But gradually this situation improved. However, for non-medical frontline workers it was very difficult to work with complete personal equipment (PPE) and maintain a social distance because of their nature of work in diverse areas and people.

“*When I went to bury Corona suspicious person's body, I wore full PPE (Personal Protective Equipment). However, when I went to the work court or other work, I used a mask, gloves, and head cover. It is tough to ensure social distance in Bangladesh due to the people's indifference. I tried but could not always maintain it*” (R9).

#### The fear of being infected by others

Because of the severity of the virus, misinformation, data, nature, and workload, the survivors experienced fear of contracting the virus. In addition, most participants feared spreading the virus to their families and close ones.

“*As I was the only earning member of my family, I always feared being affected by the virus.“ So, I tried to maintain necessary precautions like wearing a mask, washing my hands frequently, and trying to maintain distance as much as possible*” (R4).

Another participant involved in financial services (bank) closely observed a patient with COVID-19 symptoms who were not granted leave by the authorities. As a result, she feared going to the office every day. According to her statement,

“*I was afraid of going to the office regularly. One incident in my office that I want to mention here. One of our colleagues was suffering from a cold during the lockdown situation. Although she applied for leave, she was not granted it. As a result, she frequently met all of us, and we became horrified*” (R5).

### Theme II: Diagnosis

This theme centered on survivors' experiences of being infected and the diagnosis center. Three subthemes emerged from this, such as the origin of infection, physiological symptoms, and experiences in the diagnosis center.

#### Origin of infection

Those frontline workers whose duty was to provide public services and welfare amidst the severity of COVID-19 believed that they had been infected because of their frequent visits to public places and contacts.

“*The way both of us (couple) have done our professional work in dealing with the Corona situation, we assumed today or tomorrow we would be infected because we did not have the opportunity to stay at home*” (R7).

The same was true for another healthcare frontline worker who treated COVID-19 patients at a private hospital and experienced the tragic loss of three family members due to COVID-19.

“*As I was taking care of my father, like taking him to the ICU when he developed severe respiratory problems, though I was doing everything with protection, of course, I thought that I should test myself. There was a likelihood that I may have transferred the germ to my other colleagues as I was working with them in the hospital*” (R5).

#### Physiological symptoms

When the respondents were interviewed the most reported symptoms were fever, neck pain, headaches, coughs, body aches, and loss of taste and smell, as indicators of their general weakness. The comments here include examples of the earlier symptoms that the front-line workers reported.

“*I had no such symptoms except some minor neck pain. However, gradually, I developed other symptoms, and my physical situation worsened*” (R1).

“*My wife and I tested a corona examination on May 11 at Bangabandhu Sheikh Mujib Medical University with symptoms of fever, headache, slight cough, and severe pain around the ears*” (R7).

However, two respondents stated that they did not display any physical symptoms in their bodies in the initial stages of the disease or even after it was diagnosed. Even though everything about them was fine, they were listed as COVID-19-positive patients after the test.

“*Even in my case, I was certain that I was carrying the germ and infected my wife, though I had no such symptoms to be sure of my disease*” (R6).

#### Experiences in the diagnosis center

When they went to the diagnosis center for a diagnosis, those frontline employees involved in government services (such as police, magistrates, and doctors) received tremendous support. However, even though they were not required to keep queues for providing samples in the medical center, one of the respondents described his encounter with the testing facility as follows:

“*I did not need to pay any fees for my test, as it was free for all government officials. I also did not face harassment while doing tests standing in a queue or others. Instead, I went to the hospital, filled out a form with some information, gave a sample, and returned*” (R1).

However, people from lower socio-economic backgrounds who worked in the private sector struggled in the diagnosis centers. Another one commented,

“*When I went to the Upazilla health complex to do a blood test, the doctor left the hospital for having lunch*” (R3).

### Theme III: Recovery days

This theme covers a significant area of the survivors' experiences after identifying themselves as COVID-19 positive. Their initial reactions after the test report, experiences in the isolation center/home isolation, and coping mechanisms are discussed here. This theme includes earlier reactions (fear of death, depression and emotion, surprise, anxiety about family, hiding disease from family, prior mental preparation); experiences in isolation center/home isolation (care vs. denial, fear, dilemma, mental struggle, social stigma, self-isolation); and coping mechanisms (changing in diet, prayer, entertainment, telemedicine, self-realization, social support).

#### Earlier reaction

##### Fear of death

Some frontline workers experienced fear of death and anxiety after identifying themselves as COVID-19 patients. However, they informed us throughout our conversation that they were fully aware of how Bangladeshi family members and relatives consider the corpse of a corona patient as something unpleasant and unexpected.

“*When I heard it, I felt that the whole sky had broken down on me. I might die soon. Life is very short. We are just like guests on this earth*” (R1).

“*After getting the report, my whole situation changed. I became anxious. Suddenly, my hands and feet were shaking. I started thinking about death and afterward*” (R2).

##### Depression and emotion

While being identified as COVID-19 patients after the test, most of the individuals in this study reported feeling depressed. In addition, some of them have started to experience emotional instability. Even one participant with no physical symptoms of illness developed psychological weakness. The reason for this is that after the disease was reported, they were worried about the severity of the virus and its burden.

“*I was broken at first after hearing the news. Especially when Sayda hugged me and cried and said, We do not even have a baby*” (R7).

However, a 35-year-old female assistant commissioner of Kishorgonj Upazilla who initially experienced depression later revealed that she gradually turned her depression into strength.

“*I became a little depressed. However, I convinced myself that if I remained frustrated, I would die. So I had to convert my depression into mental strength*” (R4).

##### Surprisement

At least two respondents who had no physical symptoms expressed surprise when they had identified themselves as COVID-19 positive. When we interviewed them they said that they had always kept a social distance, been extremely cautious from the beginning and adhered to health regulations (including PPE). They found it difficult to believe because they were certain they had followed the precautionary measure. One of the respondents said as “*I tested only to be sure of my situation but I was astonished seeing that I was also positive while I had no symptoms*” (R6).

##### Anxiety about family

Nearly all participants admitted to becoming extremely concerned for their families after receiving confirmation of their infection. This is because they were the primary breadwinners in their household. As a result, they started to worry about what would happen to their families if the virus harmed them.

“*At that time, the first thing that came to my mind was my family, especially my younger sister*” (R2).

“*I became anxious about my family, especially about my little children*” (R9).

Another respondent who was a non-medical frontline workers had to keep his sickness a secret from his family. He was far away from them and just did not want to upset them. According to him,

“*I did not tell family members about my infection as I was far away from them. I did so because everyone was so afraid and anxious during that time of pandemic outbreak that if anyone was identified as a COVID-19 patient, people believed that that person would die soon or thought that he/she was very prone to death. So I decided not to inform my family and relatives because it could make them more sacred*” (R1).

##### Prior mental preparation

We also found a new situation after our interview. The majority of the frontline workers were panicked and became anxious, however, a 37-year-old frontline financial service provider kept her composure despite knowing she was sick. This is because, prior to becoming ill, her family had COVID-19 survivors. Therefore, she took mental preparation in advance of her infection as well as remained calm knowing the nature and severity of this virus. According to her,

“*I was not afraid. You know, it is not a dangerous disease. I was conscious only of my smell problem. However, I got used to this situation because two more people had been infected in our house. I saw them closely*” (R5).

#### Experiences in isolation

##### Care vs. denial

Most of the frontline workers we have interviewed remained in isolation at home. Only three were admitted to hospitals designed explicitly for COVID-19, where they were isolated for a minimum of 14 days. They consequently experienced positive and negative interactions with the caregivers while in isolation. However, one of the respondents, a police officer, said that the hospitals provided adequate healthcare when we asked him about his hospital experiences.

“*I was admitted to the district police hospital in Sylhet.” Moreover, three of my colleagues, who were also positive, remained in the room. The hospital authorities provided us with good treatment and everything that we required. They sent every necessary accessory as soon as we informed them over the telephone or mobile phone* (R1).

On the other hand, another non-medical frontline workers shared his bitter experiences while receiving treatment at COVID-19 dedicated hospital. He was shocked that no one, not even physicians or nurses, had ever seen him physically during his 22 days of isolation in hospitals.

“*During our 22 days of hospitalization, no doctors, nurses, or other medical staff ever visited us physically in the hospital, but only in some critical situations. Instead, they talked to us over the phone and provided only virtual advice. It frustrated me a lot to think that this is happening because we are COVID-19 patients*” (R9).

##### Self-isolation

At least two respondents described similar instances of self-isolation from family and friends. They internalized their condition and prioritized their family members' wellbeing. Even one of the responders could not go to his father's burial because he felt that other people would fall in danger of infection.

“*I have grief for not attending my father's funeral, but I took the right step at the right moment because my presence could infect others in a way that I could never accept*” (R6).

##### Fear, dilemma, and mental struggle

Almost all frontline workers experienced various forms of emotional distress in isolation, including dilemmas, insomnia, and fear of deaths. One of the frontline workers who was away from her house because of job obligations struggled to sleep and experienced death fears while alone in an isolation room at a government estate. She could not communicate with anyone because her home was kept in strict lockdown during her quarantine. She asserts,

“*Since I had trouble breathing almost every night when I went to bed, I was afraid that I might not be able to get up in the morning. I was scared to death. I thought I had these psychological problems because I was living alone*” (T2).

Another survivor, a well-known journalist, and author in Bangladesh experienced difficulty, uncertainty, and emotional stress during his mandatory home quarantine. According to him,

“*The doctor advised us to avoid social media, especially Facebook, and suggested we be busy with housework and self-care. But unfortunately, I did not listen to the doctor but once or twice used Facebook. Later, I saw that it truly created anxiety and mental pressure on me. Especially when you see that everything in the world is running smoothly, nothing is stopped because of your illness*” (R7).

Furthermore, a medical professional who cared for coronavirus patients in a private hospital in Dhaka shared similar worries with us.

“*The doctor told us to follow the routine and to become worry-free.” But nevertheless, is it possible to remain worry-free after all of this? I was shocked that even a little gastric pain was short of breath! “Corona was such a mental torture”* (R8).

##### Social stigma

The COVID-19 infection had an impact not only on the patients but also on their interpersonal and societal interactions. People purposefully avoid COVID-19 patients due to an overarching fear of infection. During their solitude, survivors allegedly experienced rejection and ostracism from those who were closest to them. At least two of non-medical frontline workers shared similar stories of denial from their colleagues and close ones when they caught COVID-19.

“*The building where I live was completely locked down. I faced some social stigma. After I got sick, I heard many officers who worked with me were terrified. Many of my colleagues around me wanted to escape. For example, after hearing the news of my infection, the naib of my office went to his village. He was afraid I might order him to work for me, but I think I have faced fewer social vulnerabilities due to my social status than other survivors*” (R2).

Even families of patients who tested positive for COVID-19 had to deal with unfavorable behaviors from their kin and neighbors Due to this stigmatization, a 38-year-old doctor who treated Corona patients in a private hospital in Dhaka had to endure severe repercussions. In his opinion:

“*As frontline workers, we had to give service directly to the COVID-19 patients. When I returned from the hospital, I understood that they feared me. However, when four other members of my family and I were infected with the COVID-19 virus, they completely avoided us*” (R6).

Another participant, a non-medical frontline workers who lost his father due to COVID-19 infection shared similar stories of being stigmatization by his neighbors. He also claimed that owing to stigma, he could not even convince anyone to hold the corpse after his father's death. The same rejection events occurred when he got infected. According to him:

“*When the villagers learned about my father's disease, they started to treat us like wild animals. Even when my father died, none of my neighbors visited our home, and there was no one to hold the corpse bed. After that, they stopped coming to our pond to take a bath when I got infected*“ (R4).

#### Coping mechanism

##### Change in diet

The majority of patients who received treatment at home altered their diet plans. They thought eating a lot would make them more prone to the illness. Some respondents stated that they also regularly engaged in breathing exercises, steamed hot baths, and sunbathing, all of which assisted their recovery from the infection.

“*According to the doctor's advice, I ate nutritious foods like milk, eggs, fish, meat, lemons, and vitamin C daily. I also took hot water vapor and followed the hygiene rules*” (R2).

##### Prayer and listening to Wazz

Bangladesh has a large Muslim population. During times of isolation, faith in God and practicing religion were very effective coping mechanisms for the survivors. Nearly everyone we had a conversation claimed that their faith in God helped them find relief from their sufferings. According to one of the respondents,

“*Most of the time of the day I prayed salah and recited dua. As I was free from all worldly activities, I concentrated more on religious activities that gave me relief* ” (R4).

Another participant who received treatment at a COVID-19-dedicated hospital regularly listened to various religious lectures (Wazz) on YouTube, which provided him with moral support to recover.

##### Music, movies, and reading books

Most of the frontline workers admitted that they avoided social media sites while remaining in isolation because it created anxiety and mental pressure upon them. One of the non-medical frontline workers asserted that he became mentally more vulnerable when he found everything around him was okay except him while scrolling into Facebook. However, most of the frontline workers passed their isolation times by watching movies, reading books, and a few of them involved in gardening.

“*I did not use social media. I read books, walked around the house, watched funny movies, and spent time gardening*” (R2).

“*Netflix and Amazon Prime helped me overcome this challenge. In series like Breaking Bad, Picky Blinders, House of Cards, Walter-Jesse's Math Lab, Celian Murphy's outstanding performance, and the political intelligence of the Underwood family, I forgot I had a fatal illness*” (R7).

##### Telemedicine

Before this pandemic, the general people of Bangladesh were not comfortable with telemedicine services. But due to the shortage of COVID-19 designated beds in public and private hospitals telemedicine services became very popular among the patients who received care remaining at home. Three individuals in our study also regularly consulted with their doctors about their health related problems over the phone, which helped them recovering from this disease. According to one of the respondents:

“*We decided to take treatment from home until the situation worsens.“ In that case, we need to be under the supervision of a specialist doctor. ”Doctor X provided us treatment via telemedicine regularly*” (R8).

##### Self-motivation

Due to the severity and concealed nature of COVID-19 at the initial stages of infection, Bangladeshi people, notably frontline workers, were more susceptible. In addition, a couple of the individuals we interviewed said that their family was their source of mental strength, aiding their recovery from this illness. One of the frontline worker who was kept in isolation in her government estate describe this situation as:

“*I have done self-counseling by convincing myself that many people depend on me. So I have to live for my siblings and my family's future. This mental strength has inspired me to recover*” (R2).

##### Social support

Almost all of the frontline workers received immense support from their family members and relatives, which helped them to recover quickly. Even if, in some instances, the family members were not physically present while they were in the hospital or home isolation, their support—such as food, comfort, and religious guidance—were tremendously helpful in enabling them to heal and cope.

“*Although I was in a COVID-19 dedicated hospital in an isolation room, I received immense support from my family. Whenever I talked with my wife over the phone, she told me not to be afraid. She motivated me in such a way that I am going to recover very soon. Even she took care of my family, children, and business in my absence*” (R4).

Some survivors also received much help from their colleagues, office managers, and friends, which was a huge help in their recovery. One of the respondents, who was admitted into a COVID-19 dedicated hospital along with four other colleagues, stated that because of their shared experiences, they could help one another and manage the situation even while remaining in a segregated unit. As (R1) said;

“*... As we were living together in a hospital room, it was great to support us all. When we felt upset, we gossiped with one another, had fun and joked, and shared our grief to maintain social distance. It worked great to recover from our psychological hardship and effectively overcome our trouble. It had a tremendous impact on us that would not have been possible if I had stayed alone in a room*.”

Although most frontline workers reported having their neighbors denied and rejected them, an author and journalist told us that his house owner offered adequate support and cooperation when the couple became infected. Due to their comparative higher social status and their connections with the power structure they were most privileged even in the age of COVID-19, but the general situation is completely different in Bangladesh. According to the respondents:

“*We informed the homeowner at the outset so that no one got panicked.” He helped us a lot from the beginning. He provided us with food, fruits, and bottled water, in addition to the constant search. He also hired a doorman who would bring emergency supplies and take down the garbage can* (R7).

### Theme IV: Post-COVID-19 days

Following their recovery, survivors also reported post-COVID-19 physiological and psychological issues, such as chest pain, sleep issues, anxiety, and shortness of breath, as well as specific social issues, such as financial loss. However, they also started to view life more optimistically. There are four subthemes under this main theme: excitement, fear and confusion, physiological problems, financial stress, and changes in philosophy.

#### Excitement, fear, and confusion

Survivors undoubtedly felt joy when they returned to normal life. Although, medical-frontline workers had to return to their previous job as there was scarcity of physicians and nurses in COVID-19 dedicated hospitals in Bangladesh. However, some participants concurred that they were afraid of spreading the virus to others after they were fully recovered. Therefore, they stayed away from places where the public congregates, like bazaars, tea shops, and playgrounds. When we questioned why they felt this way, they admitted that their test results had left them perplexed. During the pandemic, some private hospitals and clinics issued fake corona test results, which led to significant discrepancies. According to them:

“*After recovery, I tried to avoid visiting public places like bazaars, tea stalls, or friends' houses because I was scared if someone would be infected by me again. So, I tried to maintain all medical rules when communicating with others*” (R1).

“*When I fully recovered I could not believe in myself! I wanted to meet my friends and close ones. However, you know I was frightened of mixing with them. I have little trust in corona testing. So, I do not want to put anyone at risk*” (R5).

Some survivors also experienced negative attitudes from their neighbors even after their recovery from the COVID-19 virus. Despite being healed, their bodies were marked as something to be afraid of. As a result, neighbors tried to avoid them for fear of contracting an infection. According to one of the respondents:

“*Even after the report came back negative, many people stayed far away from me. So I do not know if it was for awareness or if people were scared of me*”(R2).

#### Physiological problems

The survivors experienced various physiological problems following their recovery. For instance, among the survivors breathing difficulties, respiratory issues, weakness, etc., were frequent. In addition, one survivor informed us that for a month, he could not move from one place to another owing to physical sickness.

“*Even after recovery, I often had shortness of breath. I still have some respiratory problems and have also reduced my 4 kg weight*” (R2).

#### Financial stress

Corona is a substantial financial burden on patients. Although, those frontline workers we interviewed were comparatively remained in a better socio-economic condition rather than the general population in Bangladesh, but they reported having some form of financial hardship to support their families after recuperation. One of the participants who was a doctor lost his job due to corona infection. To her surprise:

“*There has been some economic loss. The prices of daily necessities, including masks, sanitizers, fish meat, and eggs, have increased significantly. As a result, family expenses have increased immensely*” (R2).

#### Changes in philosophy

The participants returned to their everyday lives when the swab test results were reported as negative. Corona improved most of the survivors' outlooks, but a few of them who were highly educated were still uncertain about their sources of infection and the importance of mask in preventing corona virus. This is against our general beliefs about those who are illiterate are more suspicious and superstitious about the severity of corona virus. One of the non-medical frontline workers assert as: “*I do not believe a mask can protect us from corona*” (R8).

However, participants also claimed that they developed a stronger spiritual bond with God due to their COVID-19 experience. They prayed to God frequently while in the hospital and formed regular prayer routines. For example, one of the respondents who was agnostic now became a devoted follower of religion. According to him:

“*Although from the beginning I was conscientious, I was infected.” It surprised me a lot, and “I realized it was Allah's will. Therefore, I have developed a firm belief in the almighty that led me to be involved in religious rituals during my hospital days*” (R1).

## Discussion

We analyzed the lived experiences of Bangladeshi COVID-19-positive frontline workers in this study. We have identified four major themes and 13 subthemes from this study. In summary, our study showed that frontline workers in Bangladesh faced significant physiological, psychological, and social challenges while working in a new COVID-19 environment. Thus, when they contracted the infection and were isolated at home or in a hospital unit, they experienced anxiety, fear, wrath, frustration, and stigma. However, they could quickly deal with this adverse situation by altering their eating habits, relying on telemedicine for assistance, being self-motivated, having social support, and enjoying movies. The research also revealed that frontline workers continued to experience physical, emotional, economical, and social sufferings after fully recovered. In addition, when experiencing financial crisis and stigma they emphasized more on religiosity which in turn helped them to overcome those hardships. Similar themes emerged from another study on Indonesian frontline nurses who were kept in quarantine because of their infection (Siagian and Rantung, 2022).

*To begin with*, our study reveals that since the beginning of the COVID-19 pandemic, Bangladeshi frontline workers have been facing immense physical, psychological, and social challenges because of the changing working environment, their fear of infection, and separation from family and close friends. Although, they tried to maintain health protocols and social distance from the beginning, however, non-medical frontline workers told us that it was quite possible for them to maintain appropriate social distance as they had to render direct services to the general public for various reasons. Even during the period of national lockdown (started on March 23 and extended to May 30, 2020), for example, non-medical frontline financial service workers had to regularly provide banking services to the general population (Rana and Islam, 2021). As a result, among Bangladesh's frontline workers, fear of infection, trauma, distress, worry, misinformation, and social stigma are more prevalent. We also found similar findings in previous research in Bangladesh and across the world (Ahsan et al., 2021; Akhter et al., 2021; Khan Rony et al., 2021; Rahman et al., 2021; Razu et al., 2021; Sun et al., 2021; Villar et al., 2021; Wang et al., 2021, 2022; Zhang et al., 2021; Mehedi and Ismail Hossain, 2022; Pooja et al., 2022; Simeone et al., 2022; Tune et al., 2022).

*Secondly*, it was evident from the findings that most of the non-medical frontline workers were infected by this virus because of their frequent visits to different public gatherings. On contrary, medical frontline workers were reported as infected while working with the Corona unit. However, almost all the frontline workers went to the associated test centers with the physiological symptoms of fever, neck pain, headaches, coughs, body aches, and loss of taste and smell which are in line with the clinical signs of SARS-CoV-2 infection (Ramanathan et al., 2020; Wu et al., 2020). Besides, some frontline workers became very surprised that even though they had no physical symptoms, they were found positive after the test. Researchers from the University of Illinois at Chicago found similar cases. In their study Patel et al., found that of thirty five (35) infected cases, thirteen (13) never posed any symptoms (Patel et al., 2020). Moreover, due to misinformation (Islam et al., 2020; Bakebillah et al., 2021), fake COVID-19-test reports of some private hospitals (Updates, 2022), and media, some frontline workers of this study expressed their concern and confusion about the diagnosis process. In addition, most of the medical and non-medical frontline workers received adequate treatment facilities from both the test centers as well as the COVID-19 dedicated hospitals due to their upper socio-economic profiles in Bangladesh.

*Thirdly*, our study participants also revealed that fear of death, anxiety, depression, and frustration—were their immediate reactions after identifying themselves as COVID-19-positive patients. These findings are also consistent with Taylor et al. and Liu Q. et al.'s studies (Liu Q., et al., 2020; Taylor et al., 2020). From Liu et al.'s study we came to know that in Hubei, China, more than 3,000 healthcare providers were infected by the coronavirus at the initial stage which caused trauma and fear among all the frontline workers across the world. Another UK based survey study revealed that compared to the general population, frontline health care providers experienced higher levels of anxiety and depression after being infected (Murphy et al., 2020). On contrary, our study revealed that some frontline workers in Bangladesh felt less stressed as they had prior mental preparation about the severity of this virus.

Again, this study showed that most of the frontline workers we interviewed remained in isolation at home during their recovery days. Only a few went to the government's dedicated COVID-19 hospitals where they stayed in complete isolation units along with other COVID-19 patients. As there is scarcity of COVID-19 dedicated beds and people are not comfortable with remaining in isolation, like the general populations frontline workers tended to receive treatment staying at home. Similarly, in Philippines, it was found that COVID-19 survivors are more reluctant to stay at home than at the isolation center (Romulo and Urbano, 2022). The data showed that Bangladeshi frontline workers had received good care at the COVID-19 dedicated hospitals because of their upper socio-economic status. On the other hand„ some of the non-medical frontline workers were denied face-to-face treatment by the healthcare providers because of the fear of infection and stigma which is pertinent to studies conducted in India (Miah et al., 2022). Research in India also reported that some patients are stigmatized because they are COVID-19 patients (Gupta and Sahoo, 2020). As a result, some frontline workers in Bangladesh were also found to hide their disease from their friends, relatives, neighbors, and even family members when they stayed in isolation. Not only that those who received treatment at home experienced fear, anxiety, sleepiness, stress, depression, and stigmatization during their recovery days which are also consistent with a study conducted by Fawaz and Samaha (2020) over the quarantined frontline nurses in Lebanese (Fawaz and Samaha, 2020). Apart from this, some frontline workers believed that remaining in home isolation surrounded by the family members might positively impact on their recovery process. Some studies also showed that positive emotions and hope play an essential role in a patient's recovery (Carbone and Echols, 2017; Waugh, 2020).

Furthermore, frontline workers' various coping mechanisms during their recovery days are also evident in this study. Most frontline workers coped with this adverse situation by altering food habits, relying on telemedicine, religiosity, having social and organizational support, and engaging in recreational activities (e.g., music, movies, reading books). We also found that nurses in Saudi Arabia and Qatar considered changing their eating habits a solid coping mechanism against this virus (Alhusseini and Alqahtani, 2020; Villar et al., 2021). Again, like this study, studies conducted in Indonesia and the Philippines substantiated strong positive connections between religion and mental health (Romulo and Urbano, 2022; Siagian and Rantung, 2022). As most of the frontline workers in this study were Muslim, they felt a strong connection with God, which worked like a placebo effect on their healing process. Besides, some of the frontline workers mentioned that music, movies, and books were influential sources of coping strategies for stress management during isolation. The influence of music on reducing stress for quarantined COVID-19 patients is also evident in some studies (Ramesh, 2020; Carlson et al., 2021). In addition, support from family members, friends, colleagues, and neighbors were also mentioned as strong coping mechanism by the Bangladeshi frontline workers. The association between social support and healing is also consistent with previous studies (Awang et al., 2014; Siagian and Rantung, 2022; Uddin et al., 2022). Although, government of Bangladesh declared institutional supports for the COVID-19 infected frontline workers, none of the frontline workers received any organizational support of this study. Besides, no one in this study required any psychotherapy or counseling for their recovery which was evident in other countries. Even in Bangladesh, according to the survivors' experiences, strict isolation is not recommended for the survivors healing because it creates more anxiety, loneliness, and fear among the patients. Henceforth, social and organizational support could be the best coping strategies for the COVID-19 frontline workers in Bangladesh. Therefore, future researchers in Bangladesh might investigate the impact of social and organizational support on the healing process of COVID-19 survivors.

*Lastly*, the study revealed that frontline workers in Bangladesh faced several psychological and physiological challenges (e.g., breathing difficulties, respiratory issues, weakness, fear, insomnia, and fatigue) even after being fully recovered from the virus. Likewise, Guo et al., in their study, also found that fatigue, shortness of breath, fear, trauma, and stigma are common to many medical frontline workers after their recovery (Guo et al., 2022). Similar results are also found in some studies conducted in the UK and Wuhan in China (Halpin et al., 2021; Huang et al., 2021). Meanwhile, our respondents also mentioned that they also faced various social and economic crisis in their post-COVID-19 days which is also consistent with some other studies across the world (Missel et al., 2022; Rashid et al., 2022; Uddin et al., 2022).

### Strengths and limitations

This study has some limitations. Due to the spread of the virus across the nation, it was first and foremost impossible to conduct face-to-face interviews in all instances. So, again, we only conducted ten interviews, which do not represent the whole population. Earlier, we had disagreements about our study participants. Generally, healthcare providers, e.g., doctors, and nurses are considered frontline workers. However, we believe defining frontline workers is not specific but contextualized. Hence, after getting inspiration from the definition given by Rana and Islam (2021) in their recent study, we included law enforcement agencies, bureaucrats, bankers, and journalists, along with healthcare providers, as frontline workers in our study. Therefore, our study's conclusions cannot be applied to any specific group of Bangladeshi frontline workers. Despite all of these limitations, to the author's knowledge, the current study is one of the first to analyze the lived experiences of positive COVID-19 frontline workers in Bangladesh, providing in-depth detailed information on their particular experiences of suffering and coping mechanisms.

### Implications

Despite all the limitations, this paper provides comprehensive insights for the policymakers. Firstly, healthcare policymakers should design health policies based on the social setting of Bangladeshi society. In Bangladesh, there is a practice to follow and copy policies from the developed countries blindly without considering their relevance. Lockdown without a social safety net program to combat COVID-19 was not fruitful in Bangladesh. Secondly, stigma can negatively affect the victims, leading to isolation, depression, anxiety, or public embarrassment. Stigmatized individuals usually hide symptoms of their illness and restrict themselves from taking medical care. This behavior might create challenging situations to control the spread of any pandemic. Therefore, health policymakers should develop sustainable behavioral change programs to combat the social stigma associated with public health. Lastly, policymakers should develop a robust monitoring mechanism to eradicate discrimination regarding one's socio-economic status when getting medical services. This type of discrimination is a violation of constitutional rights.

## Conclusion

In any epidemic or pandemic, frontline workers suffer psychologically and socially. The lived experience of this cohort depends on various interactions between demographics and socio-economic status. Therefore, they urgently need guidance for physical rehabilitation, psychological growth, social support, and protection from social stigma. Although across the world, including Bangladesh, frontline workers' mental health conditions are studied, their everyday lived experiences as COVID-19 survivors are merely studied. This study provides a comprehensive and in-depth insight into the lived experiences of frontline workers. This study thus has other policy implications for Bangladesh. This study is significant for healthcare policymakers in planning healthcare management systems based on the social settings of Bangladesh society. This paper also suggests that national policymakers implement long-term behavioral change programs to reduce social stigma. At the same time, it suggests the government end discrimination regarding an individual's socio-economic status to get medical assistance.

## Data availability statement

Latest version of data will be available at doi:10.17632/ymwydhvztj.2.

## Ethics statement

Ethical review and approval was not required for the study on human participants in accordance with the local legislation and institutional requirements. The patients/participants provided their written informed consent to participate in this study.

## Author contributions

SA developed and designed the study, drafted the article, revised, amended the manuscript, and assumes liability in his capacity as guarantor. SA, TR, MB, MH, and MI collected and analyzed the data and provided the interpretation of the findings. TR, MB, MH, MI, and MR reviewed the paper before the submission to this journal. The final version of the paper was reviewed and approved by all the authors.

## Funding

The Bangabandhu Sheikh Mujibur Rahman Science and Technology University Research Cell (BSMRSTURC) in Bangladesh provided partial funding to the lead author for this study (Code: 3631108- FY 2020-2021: 30 June, 2021).

## Conflict of interest

The authors declare that the research was conducted in the absence of any commercial or financial relationships that could be construed as a potential conflict of interest.

## Publisher's note

All claims expressed in this article are solely those of the authors and do not necessarily represent those of their affiliated organizations, or those of the publisher, the editors and the reviewers. Any product that may be evaluated in this article, or claim that may be made by its manufacturer, is not guaranteed or endorsed by the publisher.

## References

[B1] Ahsan M. S. Ahmed S. Khan R. Hasan M. M. Kar A. Shahjahan H.. (2021). Psychological impact of COVID−19 pandemic on frontline health care workers in Bangladesh: a cross-sectional study. Bangabandhu Sheikh Mujib Med. Univ. J. 14:22–29. 10.3329/bsmmuj.v14i3.54677

[B2] Akhter S. Kumkum F. A. Bashar F. Rahman A. (2021). Exploring the lived experiences of pregnant women and community health care providers during the pandemic of COVID-19 in Bangladesh through a phenomenological analysis. BMC Pregn. Childbirth 21, 1–11. 10.1186/s12884-021-04284-5 34865620 PMC8643626

[B3] Alhusseini N. Alqahtani A. (2020). COVID-19 pandemic's impact on eating habits in Saudi Arabia. J. Public Health Res. 9, 354–360. 10.4081/jphr.2020.1868 33024727 PMC7512943

[B4] Aughterson H. McKinlay A. R. Fancourt D. Burton A. (2021). Psychosocial impact on frontline health and social care professionals in the UK during the COVID-19 pandemic: a qualitative interview study. BMJ Open 11, 1–10. 10.1136/bmjopen-2020-047353 33558364 PMC7871227

[B5] Awang M. M. Kutty F. M. Ahmad A. R. (2014). Perceived social support and well being: first-year student experience in university. Int. Educ. Stud. 7, 261–270. 10.5539/ies.v7n13p261

[B6] Bakebillah M. Billah M. A. Wubishet B. L. Khan M. N. (2021). Community's misconception about COVID-19 and its associated factors in Satkhira, Bangladesh: a cross-sectional study. PLoS ONE 16, 1–13. 10.1371/journal.pone.0257410 34506614 PMC8432838

[B7] Carbone E. G. Echols E. T. (2017). Effects of optimism on recovery and mental health after a tornado outbreak. Psychol. Health 32, 530–548. 10.1080/08870446.2017.1283039 28156138 PMC5548589

[B8] Carlson E. Wilson J. Baltazar M. Duman D. Peltola H. Toiviainen P.. (2021). The role of music in everyday life during the first wave of the coronavirus pandemic: a mixed-methods exploratory study. Front. Psychol. 12, 1469. 10.3389/fpsyg.2021.647756 34017286 PMC8129180

[B9] Dagyaran I. Risom S. S. Berg S. K. Højskov I. E. Heiden M. Bernild C.. (2021). Like soldiers on the front – a qualitative study understanding the frontline healthcare professionals' experience of treating and caring for patients with COVID-19. BMC Health Serv. Res. 21, 1–11. 10.1186/s12913-021-06637-4 34229686 PMC8260234

[B10] De Kock J. H. Latham H. A. Leslie S. J. Grindle M. Munoz S. Ellis L.. (2021). A rapid review of the impact of COVID-19 on the mental health of healthcare workers: implications for supporting psychological well-being. BMC Public Health 21, 1–18. 10.1186/s12889-020-10070-3 33422039 PMC7794640

[B11] De Simone S. Franco M. Servillo G. Vargas M. (2022). Implementations and strategies of telehealth during COVID-19 outbreak: a systematic review. BMC Health Serv. Res. 22, 833. 10.1186/s12913-022-08235-4 35764980 PMC9238134

[B12] Deng D. Naslund J. A. (2021). Psychological impact of COVID-19 pandemic on frontline health workers in low- and middle-income countries. HPHR J. 26:1–23. 10.54111/0001/Z1 33409499 PMC7785092

[B13] Farhana K. (2020). Knowledge and perception towards novel coronavirus (COVID-19) in Bangladesh. SSRN Elect. J. 6:76–9. 10.2139/ssrn.3578477

[B14] Fawaz M. Samaha A. (2020). The psychosocial effects of being quarantined following exposure to COVID-19: a qualitative study of Lebanese health care workers. Int. J. Social Psychiatr. 66, 560–565. 10.1177/0020764020932202 32489149 PMC7270571

[B15] Guo M. Kong M. Shi W. Wang M. Yang H. (2022). Listening to COVID-19 survivors: what they need after early discharge from hospital - a qualitative study. Int. J. Qual. Stud. Heal. Well-being 17:1–10. 10.1080/17482631.2022.2030001 35080475 PMC8925923

[B16] Gupta S. Sahoo S. (2020). Pandemic and mental health of the front-line healthcare workers: a review and implications in the Indian context amidst COVID-19. General Psychiatr. 33, e100284. 10.1136/gpsych-2020-100284 34192235 PMC7415074

[B17] Halpin S. J. McIvor C. Whyatt G. Adams A. Harvey O. McLean L.. (2021). Postdischarge symptoms and rehabilitation needs in survivors of COVID-19 infection: a cross-sectional evaluation. J. Med. Virol. 93, 1013–1022. 10.1002/jmv.26368 32729939

[B18] Hossain M. A Rashid M. U. B. Khan M. A. S. Sayeed S. Kader M. A. Hawlader M. D. H. (2021). Healthcare workers' knowledge, attitude, and practice regarding personal protective equipment for the prevention of covid-19. J. Multidiscipl. Healthcare 14, 229–238. 10.2147/JMDH.S293717 33564239 PMC7866910

[B19] Hossain M. B Alam M. Islam M. Sultan S. Faysal M. Rima S.. (2021). COVID-19 public stigma in the context of government-based structural stigma: A cross-sectional online survey of adults in Bangladesh. Stigma and Health 6, 123–133. 10.1037/sah0000305

[B20] Huang L. Yao Q. Gu X. Wang Q. Ren L. Wang Y.. (2021). 1-year outcomes in hospital survivors with COVID-19: a longitudinal cohort study. The Lancet 398, 747–758. 10.1016/S0140-6736(21)01755-4 34454673 PMC8389999

[B21] Islam M. S. Sarkar T. Khan S. H. Kamal A. M. Hasan S. M. M. Kabir A.. (2020). COVID-19-related infodemic and its impact on public health: a global social media analysis. Am. J. Trop. Med. Hygiene 103, 1621–1629. 10.4269/ajtmh.20-0812 32783794 PMC7543839

[B22] Khan Rony M. K. Bala S. D. Rahman M. M. Dola A. J. Kayesh I. Islam M. T.. (2021). Experiences of front-line nurses caring for patients with COVID-19 in Bangladesh: a qualitative study. Belitung Nurs. J. 7, 380–386. 10.33546/bnj.1680 PMC10367993 37496501

[B23] Khatun M. F. Parvin M. F. Rashid M. M. Alam M. S. Kamrunnahar M. Talukder A.. (2021). Mental health of physicians during COVID-19 outbreak in Bangladesh: a web-based cross-sectional survey. Front. Public Health 9, 1–7. 10.3389/fpubh.2021.592058 33634065 PMC7902057

[B24] Kwaghe A. V. Ilesanmi O. S. Amede P. O. Okediran J. O. Utulu R. Balogun M. S. (2021). Stigmatization, psychological and emotional trauma among frontline health care workers treated for COVID-19 in Lagos State, Nigeria: a qualitative study. BMC Health Serv. Res. 21, 1–13. 10.1186/s12913-021-06835-0 34419034 PMC8380097

[B25] Lai J. Ma S. Wang Y. Cai Z. Hu J. Wei N.. (2020). Factors associated with mental health outcomes among health care workers exposed to coronavirus disease 2019. JAMA Network Open 3, 1–12. 10.1001/jamanetworkopen.2020.3976 32202646 PMC7090843

[B26] Liu Q. Luo D. Haase J. E. Guo Q. Wang X. Q. Liu S.. (2020). The experiences of health-care providers during the COVID-19 crisis in China: a qualitative study. The Lancet Glob. Health 8, e790–e798. 10.1016/S2214-109X(20)30204-7 32573443 PMC7190296

[B27] Liu Y. Ning Z. Chen Y. Guo M. Liu Y. Gali N. K.. (2020). Aerodynamic analysis of SARS-CoV-2 in two Wuhan hospitals. Nature 582, 557–560. 10.1038/s41586-020-2271-3 32340022

[B28] Logie C. H. Turan J. M. (2020). How do we balance tensions between COVID-19 public health responses and stigma mitigation? Learning from HIV research. AIDS Behav. 24, 2003–2006. 10.1007/s10461-020-02856-8 32266502 PMC7137404

[B29] Magner C. Greenberg N. Timmins F. O'Doherty V. Lyons B. (2021). The psychological impact of COVID-19 on frontline healthcare workers “From Heartbreak to Hope”. J. Clin. Nurs. 30, e53–e55. 10.1111/jocn.15841 33963628 PMC8206860

[B30] Mehedi N. Ismail Hossain M. (2022). Experiences of the frontline healthcare professionals amid the COVID-19 health hazard: a phenomenological investigation. Inquiry (United States) 59:1–11. 10.1177/00469580221111925 35819056 PMC9280789

[B31] Miah M. S. Mamun M. R. Hasan S. M. Sarker M. G. F. Miah M. S. Khan M. G. U.. (2022). COVID-19 transmission flow through the stigmatization process in Bangladesh: a qualitative study. Lifestyle Med. 3, 1–7. 10.1002/lim2.52 PMC9015364 37520895

[B32] Missel M. Bernild C. Christensen S. W. Dagyaran I. Berg S. K. (2022). The marked body – a qualitative study on survivors embodied experiences of a COVID-19 illness trajectory. Scandinavian J. Caring Sci. 36, 183–191. 10.1111/scs.12975 33734468 PMC8251171

[B33] Muller A. E. Hafstad E. V. Himmels J. P. W. Smedslund G. Flottorp S. Stensland S. Ø.. (2020). The mental health impact of the covid-19 pandemic on healthcare workers, and interventions to help them: a rapid systematic review. Psychiatr. Res. 293, 113441. 10.1016/j.psychres.2020.113441 32898840 PMC7462563

[B34] Murphy J. Spikol E. McBride O. Shevlin M. Bennett K. M. Hartman T. K.. (2020). The psychological wellbeing of frontline workers in the United Kingdom during the COVID-19 pandemic: first and second wave findings from the COVID-19 Psychological Research Consortium (C19PRC). Study 44, 1–27. 10.31234/osf.io/dcynw

[B35] Nguyen T. T. Le X. T. T. Nguyen N. T. T. Nguyen Q. N. Le H. T. Pham Q. T.. (2021). Psychosocial impacts of COVID-19 on healthcare workers during the nationwide partial lockdown in Vietnam in April 2020. Front. Psychiatr. 12, 1–8. 10.3389/fpsyt.2021.562337 34354605 PMC8329079

[B36] Patel M. C. Chaisson L. H. Borgetti S. Burdsall D. Chugh R. K. Hoff C. R.. (2020). Asymptomatic SARS-CoV-2 infection and COVID-19 mortality during an outbreak investigation in a skilled nursing facility. Clin. Infect. Dis. 71, 2920–2926. 10.1093/cid/ciaa763 32548628 PMC7337684

[B37] Pooja S. D. Nandonik A. J. Ahmed T. Kabir Z. N. (2022). “Working in the dark”: experiences of frontline health workers in Bangladesh during COVID-19 pandemic. J. Multidiscipl. Healthcare 15, 869–881. 10.2147/JMDH.S357815 35496717 PMC9053477

[B38] Rahman A. Deeba F. Akhter S. Bashar F. Nomani D. Koot J.. (2021). Mental health condition of physicians working frontline with COVID-19 patients in Bangladesh. BMC Psychiatr. 21, 1–11. 10.1186/s12888-021-03629-w 34886844 PMC8655324

[B39] Rahman M. M. (2022). Social Stigma, Prejudice and Discrimination : A Study on the COVID-19 Patients and Home-Quarantined People in Barisal Metropolitan City of Bangladesh (March). Brishal.

[B40] Ramanathan K. Antognini D. Combes A. Paden M. Zakhary B. Ogino M. Planning provision of ECMO services for severe ARDS during the COVID-19 pandemic other outbreaks of emerging infectious diseases. LancetRespirMed. (2020) 8:518–26. 10.1016/S2213-2600(20)30121-1. 32203711 PMC7102637

[B41] Ramesh B. (2020). Influence of music as a coping strategy during COVID-19. SBV J. Basic, Clin. Appl. Health Sci. 3, 128–130. 10.5005/jp-journals-10082-0226634149530

[B42] Rana R. H. Islam A. (2021). Psychological impact of COVID-19 among frontline financial services workers in Bangladesh. J. Workplace Behav. Health 36, 238–249. 10.1080/15555240.2021.1930021

[B43] Rashid M. U. Khan M. A. S. Dalal K. Sagar S. K. Hossian M. Barsha S. Y.. (2022). Quality of life (QoL) among COVID-19 recovered healthcare workers in Bangladesh. BMC Health Serv. Res. 22, 1–13. 10.1186/s12913-022-07961-z 35637475 PMC9150765

[B44] Razu S. R. Yasmin T. Arif T. B. Islam M. S. Islam S. M. S. Gesesew H. A.. (2021). Challenges faced by healthcare professionals during the COVID-19 pandemic: a qualitative inquiry from Bangladesh. Front. Public Health 9, 647315. 10.3389/fpubh.2021.647315 34447734 PMC8383315

[B45] Romulo S. G. Urbano R. C. (2022). The stories of isolation and discrimination of COVID-19 sufferers in a community quarantine facility. J. Loss and Trauma 27, 92–94. 10.1080/15325024.2021.1920266

[B46] Rosenvald N. (2022). Coronavirus cases. Worldometer, 109–112. 10.26439/iusetpraxis2020.n50-51.5049

[B47] Sakib N. Akter T. Zohra F. Bhuiyan A. K. M. I. Mamun M. A. Griffiths M. D. (2021). Fear of COVID-19 and depression: a comparative study among the general population and healthcare professionals during COVID-19 pandemic crisis in Bangladesh. Int. J. Ment. Heal. Addic. 1–17. 10.1007/s11469-020-00477-9 33642957 PMC7894229

[B48] Sanders C. (2003). Application of Colaizzi's method: interpretation of an auditable decision trail by a novice researcher.. Contemporary Nurs. : J. Aus. Nurs. profession 14, 292–302. 10.5172/conu.14.3.292 12868668

[B49] Shahbaz S. Ashraf M. Z. Zakar R. Fischer F. Zakar M. Z. (2021). Psychosocial effects of the COVID-19 pandemic and lockdown on university students: understanding apprehensions through a phenomenographic approach. PLoS ONE 16, e0251641. 10.1371/journal.pone.0251641 33984059 PMC8118347

[B50] Shereen M. A. Khan S. Kazmi A. Bashir N. Siddique R. (2020). COVID-19 infection: origin, transmission, and characteristics of human coronaviruses. J. Adv. Res. 24, 91–98. 10.1016/j.jare.2020.03.005 32257431 PMC7113610

[B51] Shosha G. A. (1857). Employment of Colaizzi's strategy in descriptive phenomenology : a reflection of a researcher. Eur. Sci. J. 8, 31–43.

[B52] Siagian E. Rantung G. (2022). The experience of nurses who were isolated due to COVID-19 infection: a qualitative study. Nurs. Media J. Nurs. 12, 61–74. 10.14710/nmjn.v12i1.42239

[B53] Simeone S. Ambrosca R. Vellone E. Durante A. Arcadi P. Cicolini G.. (2022). Lived experiences of frontline nurses and physicians infected by COVID-19 during their activities: a phenomenological study. Nurs. Health Sci. 24, 245–254. 10.1111/nhs.12920 35049112

[B54] Sun P. Wang M. Song T. Wu Y. Luo J. Chen L.. (2021). The psychological impact of COVID-19 pandemic on health care workers: a systematic review and meta-analysis. Front. Psychol. 12, 626547. 10.3389/fpsyg.2021.626547 34305703 PMC8297953

[B55] Taylor S. Landry C. A. Paluszek M. M. Fergus T. A. McKay D. Asmundson G. J. G. (2020). COVID stress syndrome: concept, structure, and correlates. Depression and Anxiety 37, 706–714. 10.1002/da.23071 32627255 PMC7362150

[B56] Tune S. N. B. K. Islam B. Z. Islam M. R. Tasnim Z. Ahmed S. M. (2022). Exploring the knowledge, attitudes, practices and lived experiences of frontline health workers in the times of COVID-19: a qualitative study from Bangladesh. BMJ Open 12, e051893. 10.1136/bmjopen-2021-051893 35017240 PMC8753096

[B57] Uddin M. K. Islam M. N. Ahmed O. (2022). COVID-19 concern and stress in Bangladesh: perceived social support as a predictor or protector. Trends Psychol. 1–17. 10.1007/s43076-022-00158-7 PMC8887659 40479321

[B58] Upadhyaya D. P. Paudel R. Acharya D. Khoshnood K. Lee K. Park J.. (2020). frontline healthcare workers' knowledge and perception of covid-19, and willingness to work during the pandemic in nepal. Healthcare (Switzerland) 8, 554. 10.3390/healthcare8040554 33322486 PMC7764814

[B59] Updates L. (2022). Health Workers in Bangladesh Charged with Selling Fake Certificates'. Riyadh: Arab News.

[B60] van der Goot W. E. Duvivier R. J. Van Yperen N. W. de Carvalho-Filho M. A. Noot K. E. Ikink R.. (2021). Psychological distress among frontline workers during the COVID-19 pandemic: a mixed-methods study. PLoS ONE 16, e0255510. 10.1371/journal.pone.0255510 34351970 PMC8341539

[B61] Villar R. C. Nashwan A. J. Mathew R. G. Mohamed A. S. Munirathinam S. Abujaber A. A.. (2021). The lived experiences of frontline nurses during the coronavirus disease 2019 (COVID-19) pandemic in Qatar: a qualitative study. Nurs. Open 8, 3516–3526. 10.1002/nop2.901 33949145 PMC8242704

[B62] Wang H. Huang D. Huang H. Zhang J. Guo L. Liu Y.. (2022). The psychological impact of COVID-19 pandemic on medical staff in Guangdong, China: a cross-sectional study. Psychol. Med. 52, 884–892. 10.1017/S0033291720002561 32624037 PMC7371926

[B63] Wang H. Liu Y. Hu K. Zhang M. Du M. Huang H.. (2020). Healthcare workers' stress when caring for COVID-19 patients: an altruistic perspective. Nurs. Ethics 27, 1490–1500. 10.1177/0969733020934146 32662326

[B64] Wang H. Zhou X. Jia X. Song C. Luo X. Zhang H.. (2021). Emotional exhaustion in front-line healthcare workers during the COVID-19 pandemic in Wuhan, China: the effects of time pressure, social sharing and cognitive appraisal. BMC Public Health 21, 829. 10.1186/s12889-021-10891-w 33931034 PMC8085471

[B65] Waugh C. E. (2020). The roles of positive emotion in the regulation of emotional responses to negative events. Emotion (Washington, D.C.) 20, 54–58. 10.1037/emo0000625 31961178

[B66] World Health Organizaition. (2003). Available online at: https://www.who.int/news/item/05-07-2003-sars-outbreak-contained-worldwide

[B67] Wu X. Cai Y. Huang X. Yu X. Zhao L. Wang F.. (2020). Co-infection with SARS-CoV-2 and influenza a virus in patient with pneumonia, China. Emerg. Infect. Dis. 26, 1324–1326. 10.3201/eid2606.200299 32160148 PMC7258479

[B68] Yin Z. Zhang W. Jia X. Wang X. Hao J. Yang Y.. (2022). Psychological distress of frontline healthcare workers in the intensive care unit during the early stage of the COVID-19 pandemic: a qualitative study from China. BMJ Open 12, e049627. 10.1136/bmjopen-2021-049627 35190413 PMC8861884

[B69] Zhang X. Wang J. Hao Y. Wu K. Jiao M. Liang L.. (2021). Prevalence and factors associated with burnout of frontline healthcare workers in fighting against the COVID-19 pandemic: evidence from China. Front. Psychol. 12, 1–12. 10.3389/fpsyg.2021.680614 34484037 PMC8415624

